# 1866. Can β-lactam Combinations Restore Susceptibility Against *Mycobacterium avium* Complex: Exploring a New Approach to a “Difficult to Treat” Opportunistic Pathogen

**DOI:** 10.1093/ofid/ofad500.1694

**Published:** 2023-11-27

**Authors:** Maha Y Al-Jabri, Khalid M Dousa, Brigid Wilson, Steven M Holland, Robert A Bonomo

**Affiliations:** Case Western Reserve University - University Hospitals Cleveland Medical Center, Cleveland, Ohio; Louis Stokes Cleveland Department of Veterans Affairs Medical Center, Cleveland,, Cleveland, Ohio; VA Northeast Ohio Healthcare System, Cleveland, Ohio; National Institutes of Health, Bethesda, Maryland; Louis Stokes Cleveland Department of Veterans Affairs Medical Center, Cleveland,, Cleveland, Ohio

## Abstract

**Background:**

*Mycobacterium avium* Complex (MAC) are nontuberculous mycobacteria (NTM) responsible for chronic and debilitating diseases, with rising global prevalence. Clinical relapse/reinfection rates of MAC following treatment with first-line therapies range from 25% to 45%. β-lactam antibiotics are not utilized for the treatment of MAC due to clinically high minimum inhibitory concentrations (MICs). Recent studies have explored the effect of combining β-lactam antibiotics to reduce the MICs of other NTMs by inactivating multiple targets in the peptidoglycan synthesis pathway. Our hypothesis is that the cell wall of MAC is similar to that of other NTMs and thus, interrupting the peptidoglycan synthesis pathway using β-lactam combinations would result in lower MICs. In this study, we aim to determine the MICs of meropenem (MEM) in combination with ceftaroline (CPT), cefdinir (CDR), and cefuroxime (CXM) against MAC.

**Methods:**

A total of 31 clinical MAC isolates underwent susceptibility testing using broth microdilution method. MICs were tested for MEM, CPT, CDR, and CXM, alone, as well as combination of MEM plus either CPT, CDR, or CXM.

**Results:**

The susceptibility of MAC isolates to MEM was significantly enhanced when combined with CPT, CDR, and CXM. This effect was most prominent with the addition of CPT, with MIC_50_/MIC_90_ of < 0.125/1 µg/mL (compared to 16/64 µg/mL for MEM alone and 16/128 µg/mL for CPT alone), and CDR, with MIC_50_/MIC_90_ of < 0.125/2 µg/mL (compared to 16/ >128 µg/mL for CDR alone) (Table 1). The distribution of MICs of β-lactams alone and in combination demonstrates a “left shift” towards lower MIC values for the latter. A Wilcoxon signed-rank analysis confirmed that the change in MIC values for all β-lactam combinations compared to β-lactams alone reached statistical significance (p-value < 0.001, Figure 2).

**Figure:**
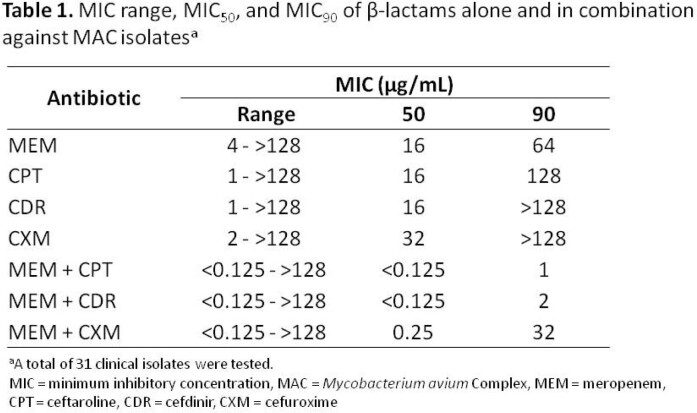

**Figure:**
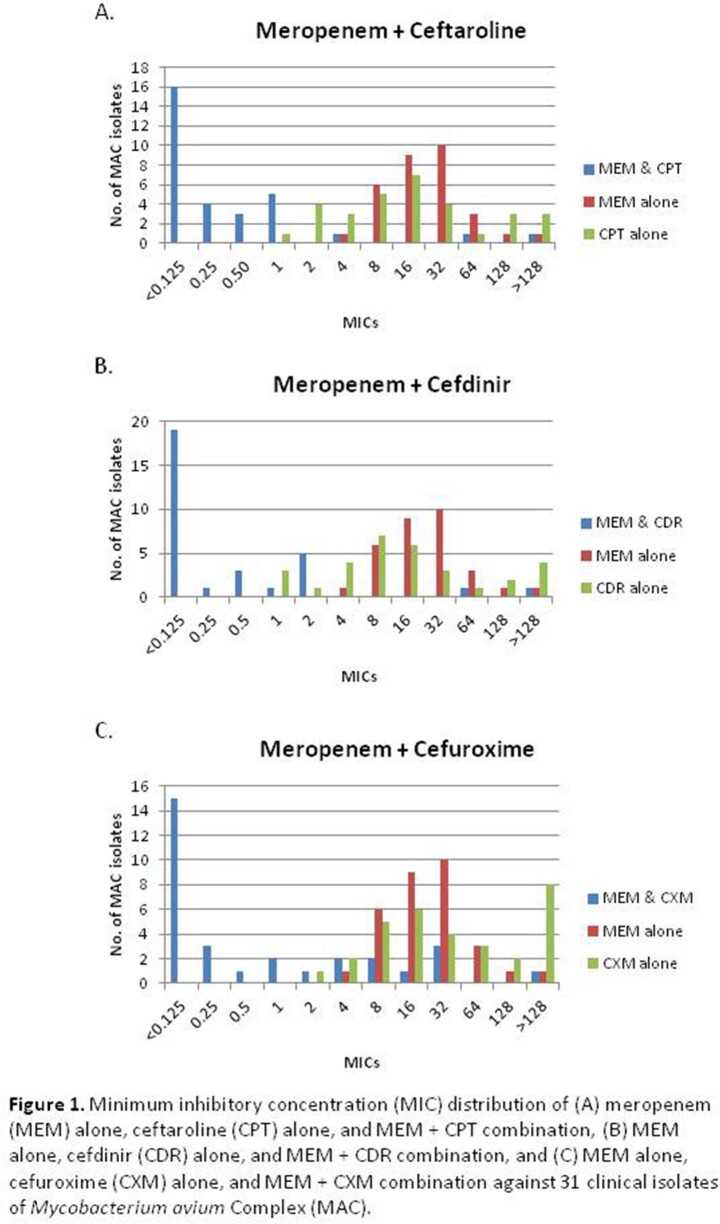

**Figure:**
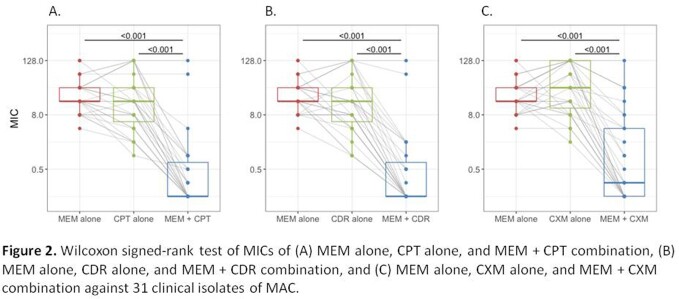

**Conclusion:**

In this study, we demonstrate that the susceptibility of MAC to MEM is restored with the addition of CPT and CDR. Our data support further exploration of β-lactam combinations as a therapeutic strategy against resistant MAC infections, refractory cases, and when first-line agents are poorly tolerated. Furthermore, our observations suggest that MAC and other NTMs share cell-wall synthetic pathways and that β-lactam combinations can overcome resistance.

**Disclosures:**

**Robert A. bonomo, MD**, Entasis, Merck, VenatoRx, Wockhardt: Grant/Research Support

